# A framework for organizing cancer-related variations from existing databases, publications and NGS data using a High-performance Integrated Virtual Environment (HIVE)

**DOI:** 10.1093/database/bau022

**Published:** 2014-03-25

**Authors:** Tsung-Jung Wu, Amirhossein Shamsaddini, Yang Pan, Krista Smith, Daniel J. Crichton, Vahan Simonyan, Raja Mazumder

**Affiliations:** ^1^Department of Biochemistry and Molecular Medicine, George Washington University, Washington, DC 20037, USA, ^2^Data Systems and Technology Jet Propulsion Laboratory 4800 Oak Grove Drive Pasadena, CA 91109 ^3^Center for Biologics Evaluation and Research, Food and Drug Administration, Rockville, MD 20852, USA and ^4^McCormick Genomic and Proteomic Center, George Washington University, Washington, DC 20037, USA

## Abstract

Years of sequence feature curation by UniProtKB/Swiss-Prot, PIR-PSD, NCBI-CDD, RefSeq and other database biocurators has led to a rich repository of information on functional sites of genes and proteins. This information along with variation-related annotation can be used to scan human short sequence reads from next-generation sequencing (NGS) pipelines for presence of non-synonymous single-nucleotide variations (nsSNVs) that affect functional sites. This and similar workflows are becoming more important because thousands of NGS data sets are being made available through projects such as The Cancer Genome Atlas (TCGA), and researchers want to evaluate their biomarkers in genomic data. BioMuta, an integrated sequence feature database, provides a framework for automated and manual curation and integration of cancer-related sequence features so that they can be used in NGS analysis pipelines. Sequence feature information in BioMuta is collected from the Catalogue of Somatic Mutations in Cancer (COSMIC), ClinVar, UniProtKB and through biocuration of information available from publications. Additionally, nsSNVs identified through automated analysis of NGS data from TCGA are also included in the database. Because of the petabytes of data and information present in NGS primary repositories, a platform HIVE (High-performance Integrated Virtual Environment) for storing, analyzing, computing and curating NGS data and associated metadata has been developed. Using HIVE, 31 979 nsSNVs were identified in TCGA-derived NGS data from breast cancer patients. All variations identified through this process are stored in a Curated Short Read archive, and the nsSNVs from the tumor samples are included in BioMuta. Currently, BioMuta has 26 cancer types with 13 896 small-scale and 308 986 large-scale study-derived variations. Integration of variation data allows identifications of novel or common nsSNVs that can be prioritized in validation studies.

**Database URL:** BioMuta: http://hive.biochemistry.gwu.edu/tools/biomuta/index.php; CSR: http://hive.biochemistry.gwu.edu/dna.cgi?cmd=csr; HIVE: http://hive.biochemistry.gwu.edu

## Introduction

Rapidly evolving sequencing technologies have exponentially increased the output of genomics data (1, 2), which has led to revolutionary discoveries in cancer biology and other biological sciences (3–5). The field of biomarker discovery has benefited tremendously from this technology, with hundreds and thousands of variations being associated with diseases from single studies (6–8). However, there are several challenges to analyzing the vast amount of data (Big Data) that next-generation sequencing (NGS) technologies are creating, and all laboratories do not have the resources to perform such large-scale studies (9, 10). Therefore, it is not surprising that many researchers still publish results from studies that involve less expensive genotyping technologies producing smaller amounts of data. Such smaller studies can sometimes help validate results from larger projects, thereby providing unprecedented levels of cooperation between scientists engaged in large- and small-scale studies.

The forementioned cooperation is difficult because genomics data are large, varied, heterogeneous and widely distributed. Extracting and converting these data into relevant information and comparing results across studies have become an impediment for personalized genomics (11). Additionally, because of the various computational bottlenecks associated with the size and complexity of NGS data, there is an urgent need in the industry for methods to store, analyze, compute and curate genomics data. There is also a need to integrate analysis results from large projects and individual publications with small-scale studies, so that one can compare and contrast results from various studies to evaluate claims about biomarkers.

Databases are mainly of two types: primary databases that comprise raw data and secondary databases that extract relationships and filter the information available from the primary databases and add annotations that are generated either manually or automatically. One of the problems often faced by end users of Big Data is the lack of curated information in primary NGS data repositories, such as NCBI Short Read Archive (12) and The Cancer Genomics Hub (https://cghub.ucsc.edu/). It is expected that curated secondary databases will help organize Big Data and make it more user-friendly, similar to what secondary databases like RefSeq (13), UniProtKB/Swiss-Prot (14) and PIR-PSD (15) have done and are still doing for GenBank (16). Coherent organization of analysis results of NGS data will also allow use of higher-level databases such as Pfam (17), PIRSFs (18), PANTHER (19), KEGG (20) and others that group objects into functional groups and provide information on biological networks and processes.

One of the major thrusts of NGS is identification of human genetic variations, which is used to better understand human diseases (21–23). Although computational approaches are available to predict which variants are potentially deleterious and associated with disease (24–27), the first steps involved in the process, such as mapping of short sequence reads to human reference and identification of single-nucleotide variations (SNVs), are computationally expensive, and few investigators have the resources or expertise to perform analysis that involves downloading terabytes of data from databases and processing and computing on them (10, 28). Furthermore, variations that are associated with cancer are currently available from diverse databases that use different workflows, and it is challenging to compare results from different sources. Many of these databases and projects have specific focus. The cBio cancer genomics portal (29) mostly consists of data from The Cancer Genome Atlas (TCGA), and its goal is to provide an integrated view of cancer genomics data from TCGA and other large projects. International Cancer Genome Consortium (ICGC) data portal allows member institutions to manage and maintain their own databases locally and also allows them to present data and information to the users through a single portal (30). UniProt provides manually curated cancer mutation data that are available from publications (14), and resources such as HGMD (31) have added to such data in the past few years. The Catalogue of Somatic Mutations in Cancer (COSMIC) (32) focuses on curating information on somatic mutations in human cancer largely from Cancer Genome Project at the Sanger Institute, UK, TCGA and other large-scale published projects. Other than UniProt, to the best of our knowledge, no group is currently engaged in extracting data through extensive manual curation of information available in publications and providing it freely. It is well known that such curation is hard to perform as expounded by Bairoch et al. in their article ‘Swiss-Prot: juggling between evolution and stability’ (33). For NGS data without the availability of clear standards in terms of data or analysis, it is even more challenging, and it is clear that not one group can tackle this challenge alone. There is a pressing need to develop data and computational standards as elegantly outlined in the recent Nature Genetic editorial (34). One of the questions posed in the editorial outlines the current state of one of the most widely used NGS pipelines ‘If I run the same sequence reads from a single cancer genome through this pipeline of assembly and variant calling twice, can I expect 70–80% concordance between the results?’ It is clear something needs to be done, and recent publications and efforts by the Human Genome Variation Society show that there is a significant interest in the research community to solve these problems (35).

In view of some of these difficulties, BioMuta has been created to integrate cancer-related non-synonymous single-nucleotide variations (nsSNVs) from various sources, which are associated with specific cancer types and publications. Such integration, we believe, will assist in the development of standards by allowing direct comparison of data provided by different groups. BioMuta is an integrated sequence feature database that provides a framework for automated and manual curation of features, such as nsSNVs. Sequence feature information in BioMuta is collected not only from COSMIC (36), ClinVar (http://www.ncbi.nlm.nih.gov/clinvar/) and UniProtKB (14) but also through active biocuration from publications and automated analysis of NGS data from sources such as TCGA using a novel data analysis platform HIVE (High-performance Integrated Virtual Environment) (37, 38). Although databases such as COSMIC add large-scale data to their databases (97 publications associated with all nsSNVs in COSMIC), our goal is to manually curate data from small-scale studies that, to the best of our knowledge, is not the focus of any of the current resources other than UniProt (118 publications associated with cancer). It is important to note that UniProt curation effort is more comprehensive than just curating cancer biomarkers; hence, we believe that our work extends the UniProt effort. We believe that computationally and manually curated and integrated data and metadata will provide unprecedented value to biological researchers by making available details from multiple studies (big and small) that ordinarily a user would not be aware of (thereby helping scientists the same way that Model Organism Databases, RefSeq, UniProtKB/Swiss-Prot and other curated databases have been doing for years).

Biocuration of data obtained from primary databases requires a framework for analyzing, annotating and computing, which has led to the development of several curation tools at all major bioinformatics institutes. Many of these biocuration tools are geared toward analysis of small-scale data such as small-number genes or proteins and therefore are not optimal for analysis of NGS data. In an elegant article ‘Big data: the future of biocuration’, Doug Howe and colleagues have pointed out how curation always lags behind data generation in funding, development and recognition (39). The authors also provide three urgent actions to tackle this problem: (i) authors, journals and curators should work together; (ii) facilitate community-based curation efforts; and (iii) support for scientific curation as a professional career. We would like to add a fourth action stating that there is an urgent need to also develop novel platforms for biocuration of Big Data. Software and hardware that have worked well for the past decades can no longer adequately support the needs of the modern curator who is analyzing vast amounts of data. In this article, we describe how time-tested curation of sequence features through reading papers supplemented with data integration from diverse sources and also through the analysis of NGS data can help create a comprehensive curated database of cancer-related nsSNVs, which can be of immediate use to the community. We subscribe to the thoughts expressed by Howe et al. that biocuration provides an organized approach in translating the recent explosion of biological data into meaningful results, and curated databases are essential for novel discoveries in biomedical research (39).

## BioMuta data sources

**Figure 1.**
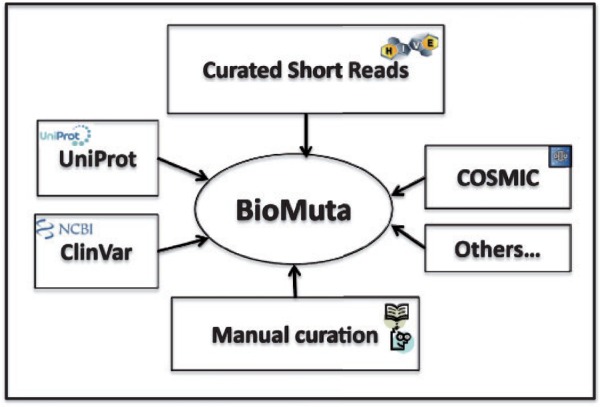
nsSNV data from various sources are collected, filtered and mapped to UniProtKB/Swiss-prot–defined complete human proteome and integrated into BioMuta.****

Data sources for BioMuta are shown in Figure 1. Unless otherwise noted, all accessions and identifications (IDs) are mapped using ID Mapping table (40), followed by pairwise alignment and mapping of sequence positions with methods that have been used previously (24, 41). Only those nsSNVs that could be mapped to UniProtKB/Swiss-Prot human protein that have the Complete Proteome keyword tag are retained in BioMuta.

Although there are several efforts worldwide to collect and disseminate cancer genomics variation data, it is clear that the data are heterogeneous and it is difficult for users to compare and contrast data from different data sources. Different algorithms are used to identify variations, and also, to the best of our knowledge, biocuration of variation data from publications on cancer biomarkers is limited. In all, 118 publications were retrieved from UniProt and 97 from COSMIC. The BioMuta project through literature mining-assisted curation has already added 85 publications that are not present in either COSMIC or UniProt. In addition to this, the complementary Curated Short Read archive (CSR) project provides additional mutation data to BioMuta from TCGA. Future plans include addition of data from ICGC and other cancer genomics projects as data linked to publications becomes available from these resources (criteria for inclusion of external data in BioMuta include association of record with a publication).

### Catalogue of Somatic Mutations in Cancer

The file, CosmicWGS_MutantExport_v65_220513, which contains all coding point mutations, was downloaded from COSMIC (42). The first step involved filtering out entries without a PubMed identification (PMID). Because the cancer descriptions in COSMIC are complex, the cancer description columns (primary site, site subtype, primary histology and histology subtype) were manually checked and converted into TCGA cancer categories (https://tcga-data.nci.nih.gov/tcga/tcgaHome2.jsp). A total of 283 895 nsSNVs of 904 143 variations were retrieved from the COSMIC file.

### ClinVar

ClinVar (http://www.ncbi.nlm.nih.gov/clinvar/) is a database that provides information about sequence variations and associations to human health. Tables were downloaded from the ClinVar ftp site (ftp://ftp.ncbi.nlm.nih.gov/pub/clinvar/). A total of 4590 cancer-related variations with PMIDs were retained. The majority of the records from ClinVar were filtered out because they either did not have PMID or the cancer type was not easily discernible.

### UniProtKB

UniProt (14) provides comprehensive curated protein sequences and functional information. All proteins that contained cancer-related keywords (cancer, carcinoma glioma, blastoma, leukemia, melanoma, adenocarcinoma, lymphoma and tumor) in the sequence feature (FT) line were extracted from UniProtKB/Swiss-Prot–defined human complete proteome, and 2279 manually verified variations were added to BioMuta. UniProt does not provide genomic location; hence, for these variations genomic locations are not provided.

### Manual curation

By using key terms [cancer, single-nucleotide polymorphism (SNP), biomarker, variant, variation, etc.], articles from PubMed (http://www.ncbi.nlm.nih.gov/pubmed) were retrieved and manually curated to obtain variation information. PMIDs not present at the time of curation in COSMIC, ClinVar and UniProtKB were selected for manual curation. A total of 139 sites from 85 articles were added to BioMuta through this process.

### Curated Short Read archive

Currently there are thousands of large-scale NGS data from patients and cell line samples that are available from primary short read data repositories such as TCGA (http://cancergenome.nih.gov/) and NCBI Short Read Archive (43) and listed through dbGaP (44). We expect that integrated analysis of these data will lead to novel discoveries. For example, NGS data from TCGA provides a rich source of sequence data that can be mined to extend and complement mutation and SNV information available from dbSNP, UniProt, COSMIC and other variation databases. We intend to identify all nsSNVs from representative samples from all data sets that have matched case and controls and also have exome and RNA-Seq data. Analysis of these subsets of samples provides a rich source for biological discovery. All variation data can be further analyzed using SNVDis, which is a proteome-wide SNV distribution analysis tool (24). For this study, NGS data from 20 breast cancer patients (22 tumor samples and 33 normal) were analyzed to identify nsSNVs. Results from this analysis and additional information such as phenotypic information were curated and added to a CSR. A total of 31 979 nsSNVs of 291 803 SNVs from tumor samples were added to BioMuta. Direct access to CSR is available at http://hive.biochemistry.gwu.edu/dna.cgi?cmd=csr. Users can search for variations present in tumor and normal samples using gene or protein accession numbers and view whether the variation is already present in dbSNP. Searching using TCGA IDs is also supported. The CSR curation platform is supported by HIVE, which is described in the section below.

### HIVE for biocuration

A sophisticated IT framework is required for analyzing, annotating and computing the vast amounts of data generated using NGS technologies. HIVE provides such a platform and is used to analyze NGS data. HIVE is a bio-computing operating system, which provides the ideal backbone to integrate modular software into a data analytics backbone. The HIVE architecture provides a highly parallel processing environment, which allows optimal compatibility and performance for both native and industry-standard tools. All algorithmic services and tools manipulate data from three sources: data loaded preliminary into the system, data provided by the user during a computation inquiry or data computed during a previous computation. HIVE has an ensemble of parsers, loaders, converters and validators for all industry-standard biological data formats (such as sequences, alignments, profiles). All data in the system are available for downloading in a variety of industry-accepted data formats (fasta, SFF, fastq, BAM, SAM). The primary step in many genomic workflows is to align and map short reads to a reference genome. There are several software programs with their own alignment algorithms. The different algorithmic approaches of each tool create computational trade-offs in speed, accuracy and performance to optimize the detection of variants in the alignment (45, 46). Currently, HIVE has the following alignment tools integrated and parallelized: HIVE-hexagon (native HIVE alignment tool), Bowtie (47) and BWA (48). After alignment of short reads to a reference genome with any of the alignment tools, variants can be identified through comparison of the sample genome with the reference genome.

**Figure 2.**
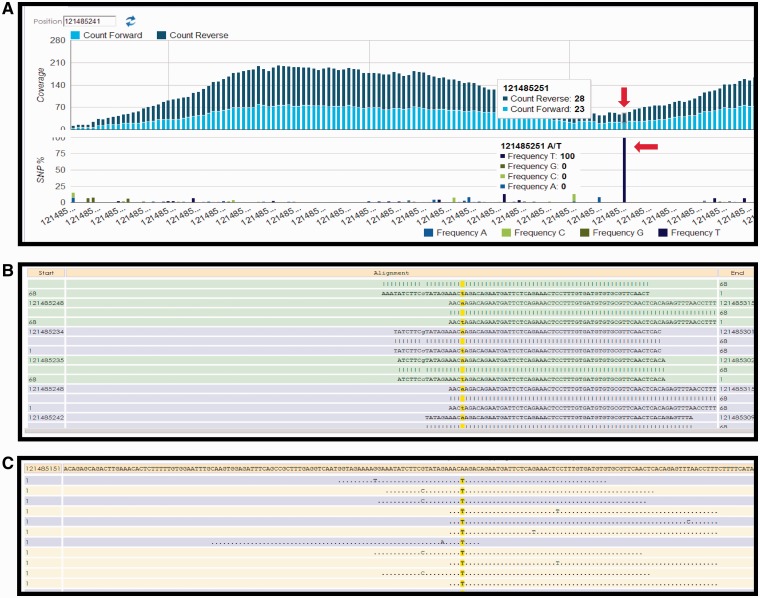
HIVE interface showing result obtained from SNV profiling of short sequence reads mapped to nucleotide sequence surrounding a variation site. (**A**) Overall coverage result with the 121 485 241 position, showing variation. (**B**) Reads mapped to the reference sequence with the column of interest are highlighted in yellow. (**C**) Only variations are shown in this panel.

Currently, the following protocol is in use in the CSR project (a BioMuta data source) to identify variations: Short read data are obtained from TCGA (http://cancergenome.nih.gov/) via The Cancer Genomics Hub data portal (https://cghub.ucsc.edu/). The reference used in the alignment is the hg19, GRCh37 Genome Reference Consortium Human Reference 37 (GCA_000001405.1) downloaded from UCSC (http://hgdownload.cse.ucsc.edu/goldenPath/hg19/chromosomes/). UniProtKB protein amino acid position and ID mapping is done using SNVDis and ID Mapping services (24, 40). After the raw SNV data are generated using Bowtie (47) and SAM tools (49), filters are used to select high-quality SNVs that are of desirable coverage (>10 reads) and quality score (>20). The filtration process also rejects detected SNVs falling out of the exome regions. Results of the variation profiling tool can be further evaluated manually using HIVE native displays as shown in Figure 2.

## BioMuta content

To ensure usability of the database, care is taken to verify that all SNVs in BioMuta have the following characteristics: (i) has PMID for all imported data, (ii) is an nsSNV, (iii) can be mapped to UniProtKB/Swiss-Prot–defined human proteome and (iv) has either gene/protein or genome coordinates. SNVs associated with PMIDs that report <1000 variations are considered small-scale study variations, and those that are associated with PMIDs that report >1000 variations are considered large-scale studies and are hence marked as large-scale study variation. Literature mining variations are those that are automatically extracted through literature mining procedures. Such variations are currently not available to the public. Table 1 provides detailed statistics of the number of variations obtained from different databases. The majority of the variations are obtained from COSMIC and CSR-TCGA. Through manual curation of 85 publications, 139 sites were added to BioMuta. Adding such manually curated records in BioMuta is one of the top priorities of the project. Table 2 provides an overview of example search parameters, number of articles retrieved and overall number of articles that were found to include variation information that can be included in BioMuta. More specifically, different combinations of search terms are used and multiple search results are combined to create a nonredundant set of PMIDs. Title and abstracts are read to extract articles of interest. Titles/abstracts with gene/protein name, cancer type, tumor type, variation site, mutation- and biomarker-related words are prioritized for curation. The next step involves reading the manuscript and any relevant Supplementary Tables to retrieve variation-related information. Finally, accession numbers, mutation and mutation positions are verified, and attempts are made to manually check and include missing information such as chromosomal location, accession number/s and valid HGNC gene symbols. Curation results are cross-checked by curators and through the validation process.

**Table 1.** Twenty-six cancer types and 322 882 (small-scale: 13 896; large-scale: 308 986) associated variations in BioMuta

Cancer types^a^	COSMIC		UniProt		ClinVar		Manual		CSR-TCGA	
	Small- scale^b^	Large- scale^c^	Small- scale	Large- scale	Small- scale	Large- scale	Small- scale	Large- scale	Small- scale	Large- scale
Lung (LUAD)	121	80 006	105							
Colon (COAD)	486	68 249	235				20			
Breast (BRCA)	176	7386	342	1		3314	16			31979
Esophageal (ESCA)	43	25 980					1			
Ovarian (OV)	1229	16 411	31			1276	4			
Skin (SKCM)	496	17 041					2			
Prostate (PRAD)	77	10 920					1			
Head and neck (HNSC)	716	11 838					1			
Rectum (READ)		9760					10			
Lymphoid (DLBC)	1710	7006								
Adrenocortical (ACC)	1000	4515		1						
Pancreatic (PAAD)	896	3164	3							
Brain (LGG)	773	2383								
Uterine (UCEC)	490	1414	1							
Kidney (KIRC)	893	115								
Liver (LIHC)	1224	1023	14				3			
Glioblastoma (GBM)	776									
Acute myeloid (LAML)	409						8			
Thyroid (THCA)	513		7				3			
Bladder (BLCA)	450		2							
Lung (LUSC)		256								
Stomach (STAD)	89									
Kidney renal (KIRP)	33		42							
Kidney chromo (KICH)	57									
Non-small lung (NSCLC)			4				8			
Cervical (CESC)			1				5			
Other^d^	5319									

^a^LUAD, lung adenocarcinoma; COAD, colon adenocarcinoma; BRCA, breast invasive carcinoma; ESCA, esophageal carcinoma; OV, ovarian serous cystadenocarcinoma; SKCM, skin cutaneous melanoma; PRAD, prostate adenocarcinoma; HNSC, head and neck squamous cell carcinoma; READ, rectum adenocarcinoma; DLBC, lymphoid neoplasm diffuse large B-cell lymphoma; ACC, adrenocortical carcinoma; PAAD, pancreatic adenocarcinoma; LGG, brain lower grade glioma; UCEC, uterine corpus endometrial carcinoma; KIRC, kidney renal clear cell carcinoma; LIHC, liver hepatocellular carcinoma; GBM, glioblastoma multiforme; LAML, acute myeloid leukemia; THCA, thyroid carcinoma; BLCA, bladder urothelial carcinoma; LUSC, lung squamous cell carcinoma; STAD, stomach adenocarcinoma; KIRP, kidney renal papillary cell carcinoma; KICH, kidney chromophobe; NSCLC, non-small cell lung cancer; CESC, cervical squamous cell carcinoma and endocervical adenocarcinoma.

^b^Small-scale—SNVs associated with publications that report <1000 SNVs.

^c^Large-scale—SNVs associated with publications that report >1000 SNVs or SNVs identified using computational pipelines from existing NGS data.

^d^Cancer types not specified or well defined.

**Table 2.** Example PubMed search terms and results

Search terms	Total articles^a^	Positive articles^b^
SNP, biomarker, cancer	702	60
Biomarker, cancer, single-nucleotide- polymorphism	1986	43
Polymorphism, biomarker, cancer	5215	20
SNP, exon, cancer	394	16
Gene name^c^, cancer, SNP	20	4
Total		143^d^

^a^Total number of articles retrieved using the search terms.

^b^Articles from which data were extracted for inclusion in BioMuta.

^c^Targeted curation of specific genes, e.g. MTA1, MTA2, SULF2, SHBG, DLX4, etc.

^d^Articles and annotations that pass validation step are retained.

Users can download the entire BioMuta table or browse the database by searching for records using gene names and UniProtKB or RefSeq accessions. Search results include a graphical representation of the mutations and a table that can be downloaded in tab-delimited format and further analyzed by Microsoft Excel or simple scripts. Users have the ability to select a specific row in the results table and send comments to BioMuta curators. This type of direct feedback will help us improve database content. All records are linked to the Early Detection Research Network (EDRN) Knowledge Environment through its online public portal (50). EDRN is a distributed knowledge network that integrates cancer biomarker research results from across the network. This includes the integration of annotations regarding biomarkers under study with the results from those studies that can be used for analysis. The biomarkers themselves are annotated from studies performed by the EDRN and linked to the publications and external protein and genomic databases. The annotations include information about the success of the biomarkers that have been studied. The EDRN Knowledge Environment allows for external linking to the specific data captured within the system. This has allowed for BioMuta and EDRN to be linked together through specific attributes of the biomarkers, including common gene names provided by HGNC (51), which are annotated with the biomarkers within the EDRN knowledge system and biomarker database. The integration of these highly curated systems becomes plausible given the adoption of common identifiers and the promotion of online portals and web services.

BioMuta has data from various sources, and it is possible that some of these databases might contain errors in terms of the genomic coordinates and/or the gene/protein positions. To reduce the propagation of these types of errors, we have validation procedures to check the table. To address the heterogeneity of the different variation data sources, all variant records are unified via the UniProtKB/Swiss-Prot human proteome set by providing each variant a UniProtKB accession number and position. To achieve this, all variations with genomic coordinates are first mapped to genes/transcripts using SeattleSeq services (http://snp.gs.washington.edu/SeattleSeqAnnotation137) and then mapped to UniProtKB protein accession and position using methods described previously (24). Briefly, the mapping process includes mapping of RefSeq accessions to UniProtKB accessions using ID mapping services provided by Protein Information Resource and UniProt (40), followed by pairwise alignment of the sequences to map the positions. For all the records that cannot be correctly mapped to the coding region or if the amino acid does not match the UniProtKB-defined proteome or for nucleotides if they do not match the RefSeq nucleotide for that position, the entire row is discarded after manual evaluation of the error.

## BioMuta utility

**Figure 3.**
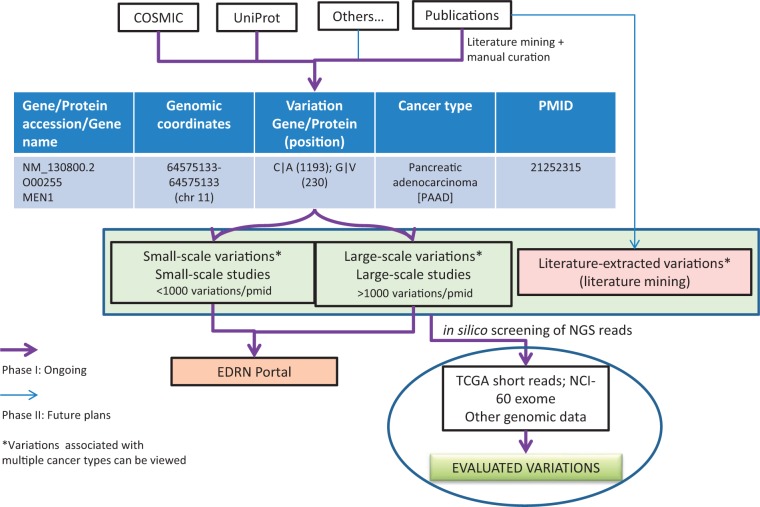
BioMuta data flow and utility in evaluating variations obtained from various cancers.

One of the immediate applications of the BioMuta project is to evaluate variations that are obtained from various sources through biocuration and thereby provide ways to prioritize variations for further experimental evaluation by the EDRN community and others. The evaluations can be performed by both comparing and contrasting mutations data from different cancer types and/or from different studies. Additional evaluation of mutations can also be performed by interrogating NGS data from TCGA or ICGC or other projects to see whether specific mutations are present in certain cancer types and what their frequency is (Figure 3).

**Figure 4.**
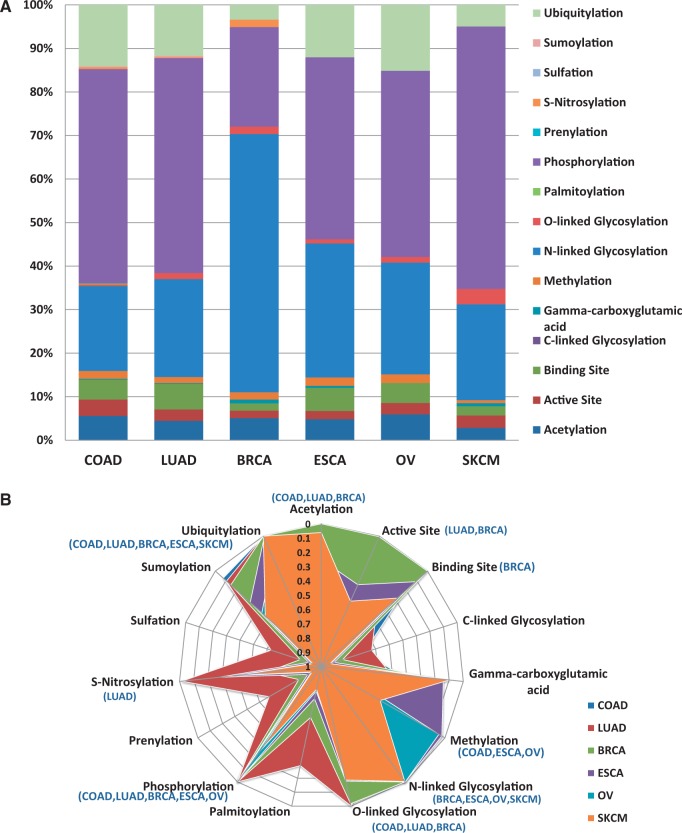
Loss of functional sites (PTM sites, active and binding sites). (**A**) Top six cancer types with the highest number of records in BioMuta. Lung adenocarcinoma (LUAD), colon adenocarcinoma (COAD), breast invasive carcinoma (BRCA), esophageal carcinoma (ESCA), ovarian serous cystadenocarcinoma (OV) and skin cutaneous melanoma (SKCM). (**B**) Statistical analysis of loss of functional sites show that for some cancer type–specific functional sites are less susceptible to variation (colored graph area almost touching the perimeter—where perimeter represents *P*-value close to 0).

One of the goals of researchers is assessing the functional impact of variations. Figure 4a provides example analysis results of how the BioMuta data can be used to better understand the functional impact of nsSNVs from different cancer types. For this analysis, all the nsSNVs were mapped to functional sites that were obtained from UniProtKB sequence feature annotation. Based on this analysis, we notice that a large number of posttranslational modifications (PTMs) and active and binding sites are affected by nsSNVs. It is interesting to note that for breast cancer, there is a high number of nsSNVs that affect N-linked glycosylation sites. To find out whether certain types of PTM or other functional sites are resistant to variations, *P*-values were calculated based on methods described earlier (24, 52), to estimate the significance between observed and expected numbers. The results indicate, as expected, for several of the functional sites, observed variations are significantly lower than the calculated expected values. More specifically—acetylation: observed 105, expected 224.28, *P*-value 4.53E-19; active site: observed 59, expected 93.18, *P*-value 9.89E-05; binding site: observed 102, expected 123.69; C-linked glycosylation: observed 1, expected 1.68; γ-carboxyglutamic acid: observed 5, expected 2.61; methylation: observed 45, expected 26.09, *P*-value 2.42E-07; N-linked glycosylation: observed 551, expected 467.78, *P*-value 9.60E-05; O-linked glycosylation: observed 28, expected 76.64, *P*-value 1.54E-10; palmitoylation: observed 1, expected 4.52; phosphorylation: observed 1083, expected 2325.07, *P*-value 1.71E-183; prenylation: observed 0, expected 2.00; S-nitrosylation: observed 7, expected 22.25, *P*-value 1.65E-04; sulfation: observed 1, expected 1.68; sumoylation: observed 7, expected 19.19, *P*-value 1.33E-03; ubiquitylation: observed 257, expected 668.12, *P*-value 3.78E-74 (*P*-values >0.05 are not shown). Data from specific cancer types were also analyzed to evaluate whether certain PTMs are more affected by certain types of cancer (Figure 4b). The majority of functional sites analyzed seem to be protected from mutation (significantly less observed variations than expected). It is hard to explain why for certain cancer types some of the functional sites appear to be less protected. More data are required to evaluate these trends. All the variations obtained in our pipeline are also integrated into SNVDis (24). To facilitate evaluation of the effects of variations, PolyPhen-based (6) predictions are also included in the BioMuta table. SNVDis provides users with applications that can be used to evaluate the distribution of nsSNVs on protein functional sites, domains and pathways at the entire proteome level. Such proteome-wide analysis is complementary to functional impact analysis using methods such as PolyPhen (27) and SIFT, (26) and similar algorithms.

Integration of data, as the one performed in this study, allows identification of genes that have high level of variations. From the small-scale category, the top five genes in terms of number of unique nsNSVs–PMID pairs are TP53, PBRM1, MEN1, ARID1A and NF1. For the large-scale category, the genes are BRCA2, BRCA1, TP53, TTN and CACNA1C. In search of variants that are recorded in more than one database, the variants that have same UniProtKB accession, amino acid position and variation were identified. There are 518 variants found in two or more data sources (Supplementary Table S1). We expect this overlap to increase as more data from other cancer-genomics studies are included. Of these 518 overlapping variants, the CSR database contributes the most to this number of shared variants (>95%), thereby showcasing the utility of evaluating variations by analyzing TCGA data. Another interesting fact is that almost 90% (23 of 26) of the literature-based manually curated variation overlaps are from CSR and not from COSMIC, suggesting that CSR database even with a limited number of patient data might already be useful in evaluating published cancer-related variations.

Our overarching goal is to provide whole-genome and exome analysis capabilities through HIVE or similar platforms where users can upload short read sequences and map them to the human reference genome followed by flagging of sites that are impacted by variations and are already reported by other studies. Such analysis will allow researchers to quickly evaluate personal genomes of patients or study subjects.

## Future perspective

Efforts directed toward creating databases such as CSR, ClinVar, RefSeq, UniProt, HGMD (31) PharmGKB 54), IntOGen (7), ICGC (8) and others, which provide information on variations and disease or other phenotypic details, will provide methods connecting genomic alterations with clinical parameters. These efforts are vital for using the full potential of NGS technologies (3), leading to novel discoveries that will translate to diagnostic and therapeutic targets (4, 54). All of these databases will benefit from additional variation sites extracted through the biocuration of information from publications. Our future plans include automated literature mining methods that will provide targeted extraction of publications that can be used to annotate major cancer genes. We also intend to provide community annotation tools to cancer biologists so that they can add notes related to experimental validation of the variations and the possibility of using them as diagnostic or prognostics markers. This information can be used by curators to provide additional structured information to these entries. Engaging the entire scientific community in community annotation efforts has been difficult (51, 55). Therefore, we will initially focus on involving the EDRN community and other specific cancer researcher groups such as Alliance of Glycobiologists (http://glycomics.cancer.gov/) in our initial community annotation efforts.

## Access

BioMuta and CSR are updated at least once every 4 months. Access to all data is available without any login requirements. To use HIVE’s computationally intensive tools, users need to register at http://hive.biochemistry.gwu.edu/dna.cgi?cmd=userReg. Temporary login is provided for evaluation purposes such as browsing the interfaces or viewing example analysis results. HIVE login URL: http://hive.biochemistry.gwu.edu/dna.cgi?cmd=login&follow=home; evaluation userid: xlhive@yahoo.com; password: pilotHive5. Users can also install HIVE on their own hardware or use HIVE-in-a-box, which is a low-cost alternative to analyze NGS data using predetermined workflows. For additional details, users are encouraged to contact the HIVE team (http://hive.biochemistry.gwu.edu/dna.cgi?cmd=contact).

## Supplementary Data

Supplementary data are available at *Database* Online.
